# Supplementary figures and images for: Susceptibility to disease (tropical theileriosis) is associated with differential expression of host genes that possess motifs recognised by a pathogen DNA binding protein
Source: PLoS One. 2022 Jan 21;17(1):e0262051. doi: 10.1371/journal.pone.0262051 (PMC8782480; doi:10.1371/journal.pone.0262051)

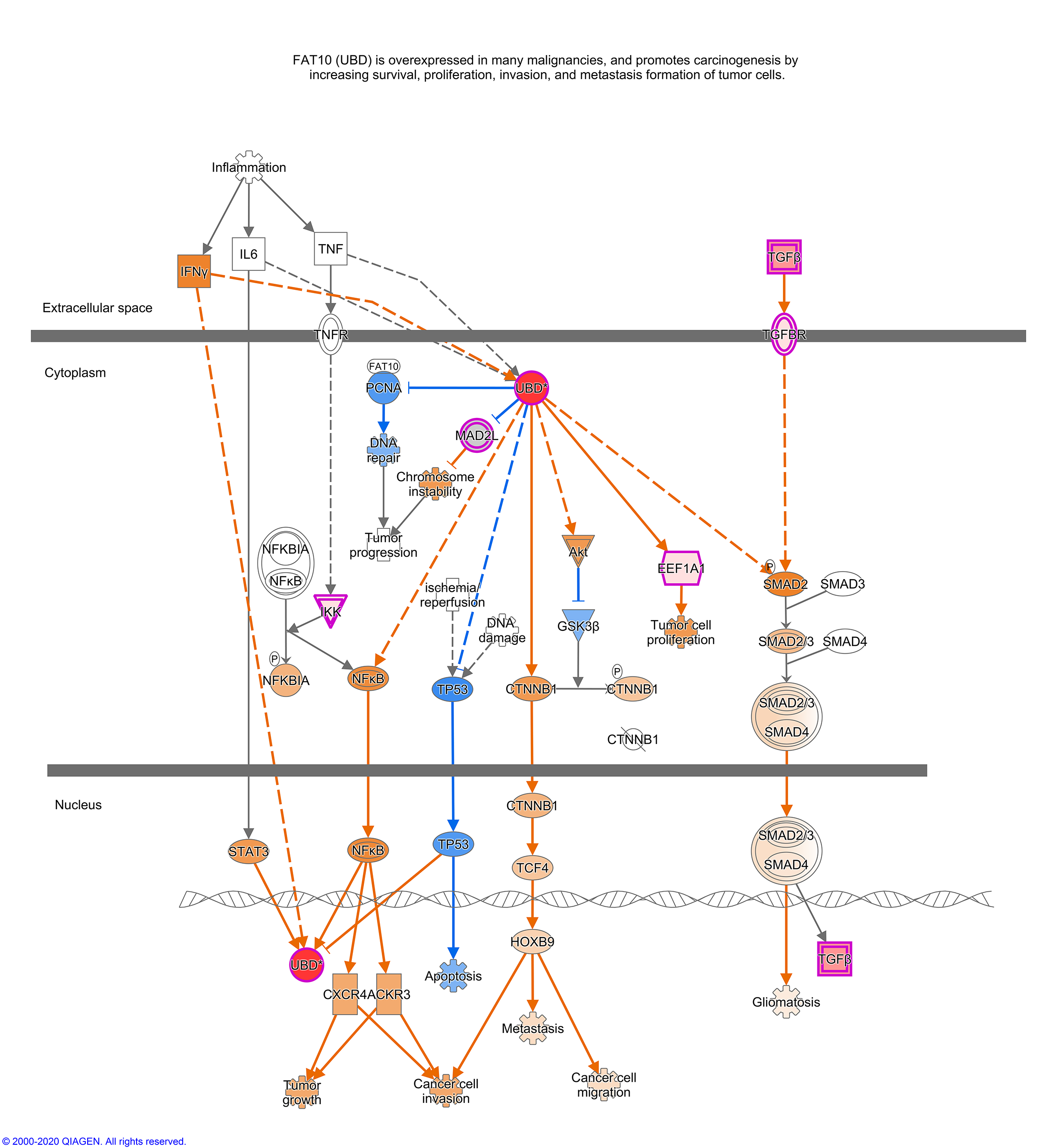

Supplement: S1 Fig — The nodes represent genes/molecules/complexes in a pathway, and the lines and arrows between nodes indicate known relationships from the Ingenuity Knowledge Base. Nodes with purple outline indicate molecules that were measured as differentially expressed in our dataset, with the intensity of coloured infill indicating the level of up–(red) or down-(green) regulation of Holstein relative to Sahiwal. The blue and orange coloured molecules and lines are predicted activation states generated by the Molecular Activity Predictor function in Ingenuity Pathway Analysis. Blue colour indicates a predicted inhibition, and orange a predicted activation state in Holstein relative to Sahiwal. Broad lines with explanatory text beside the pathway indicate the cellular location of molecules in the pathway. The molecules in the pathway are given shapes that indicate their functional class (Nested Circle/Square = Group/Complex, Horizontal ellipse = Transcriptional Regulator, Vertical Ellipse = transmembrane receptor, Vertical Rhombus = enzyme, Square = Cytokine/Growth Factor, Triangle = Kinase, Vertical Ellipse = Transmembrane Receptor, Circle = other). The edges between molecules are also differentiated to indicate the type of relationship between them. Solid lines are direct relationships and dashed lines are indirect. (TIF) [file pone.0262051.s001.tif]

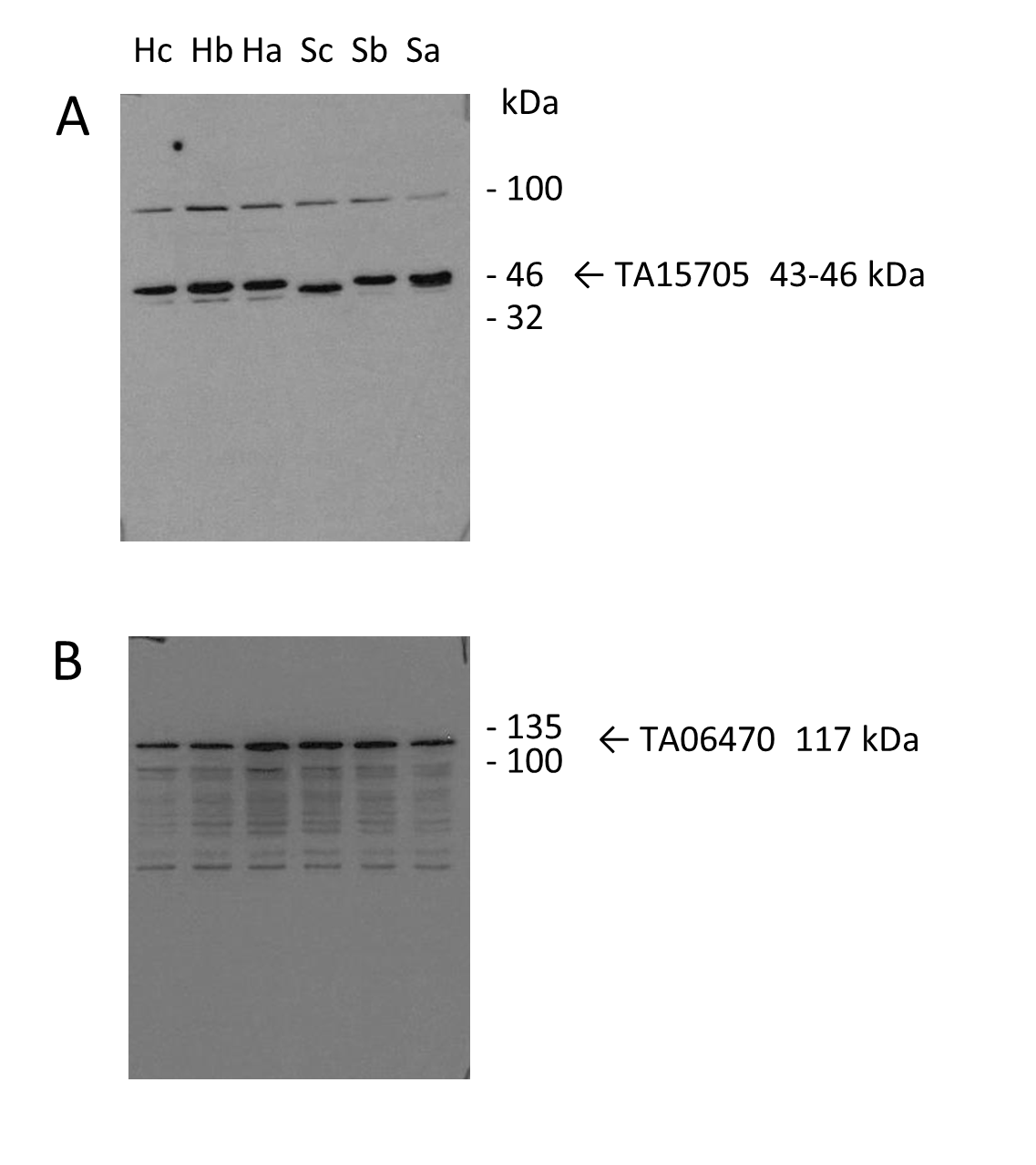

Supplement: S2 Fig — Sahiwal samples are denoted SA, SB, SC; Holstein denoted HA, HB, HC. Protein size markers are indicated on the right (kDa). A. Extracts probed with Rat anti-Ta9 (TA15705) at 1/1200 dilution. Ta9 was detected at a variable size of 43-46kDa, which is a similar to that described previously for the polymorphic Ta9 antigen [17,43]. B. As a control, the same blot was reprobed with Rabbit antiserum raised against constitutively expressed ER HSP90 (TA06470) at 1/1500 dilution. The Ta9 and HSP90 reactive proteins are denoted by arrow. (TIF) [file pone.0262051.s002.tif]

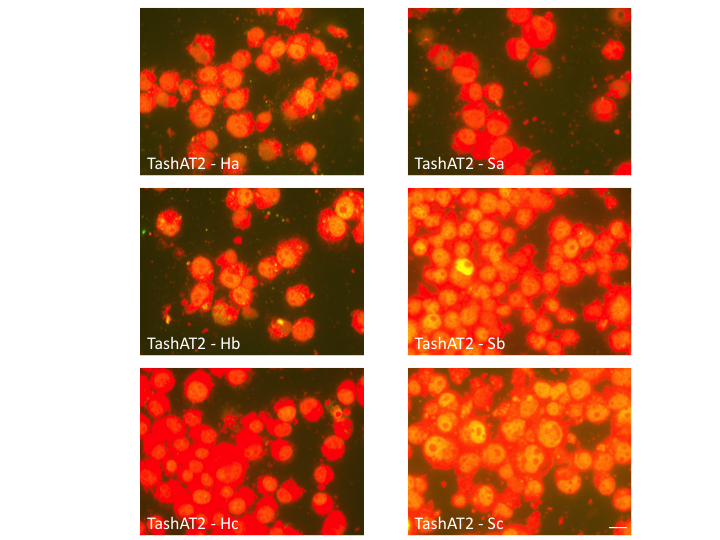

Supplement: S3 Fig — Sahiwal samples are denoted SA, SB, SC; Holstein denoted HA, HB, HC. Cells were reacted with antiserum specific for TashAT2 (EL24) and images obtained using matched exposures. Bar = 7 μm. (TIFF) [file pone.0262051.s003.tiff]
