# Supplementary material for: Susceptibility to disease (tropical theileriosis) is associated with differential expression of host genes that possess motifs recognised by a pathogen DNA binding protein
Source: PLoS One. 2022 Jan 21;17(1):e0262051. doi: 10.1371/journal.pone.0262051 (PMC8782480; doi:10.1371/journal.pone.0262051)
Supplement: S1 Table — F and R denote forward and reverse primers respectively. *Primers designed based on Ensembl transcript. (DOCX) [file pone.0262051.s004.docx]

| **Gene** | **Accession**  **No.** | **Orientation** | **Primer sequence (5’-3’)** |
| --- | --- | --- | --- |
| Interferon alpha (IFNA) - generic | NM_001172040-2 | F  R | AAGCCATCTCTGTGCTCCAC  CCCCTCCTCCTGCCTCAG |
| Interferon beta 1 (IFNB1) | NM_174350 | F  R | TGAGGAGATGAAGCAAGAACAG  GGTGAGAATGCCGAAGATGT |
| Interferon beta 3 (IFNB3) | NM_001114297 | F  R | AGCCCTGTGCCTGTTTTCATCA  CCGCCTTTGCTGGAATCTGAG |
| Interleukin 6 (IL6) | NM_173923 | F  R | ACCACTCCAGCCACAAACAC  ATGCCCAGGAACTACCACAA |
| Interleukin 23A (IL23A) | XM_588269 | F  R | AGACCTCCGTGGCTCAGTAA  TCAACATCGTCAGTCAGTCAGT |
| ISG15 ubiquitin-like modifier (ISG15) | NM_174366 | F  R | GATCAATGTGCCTGCTTTCC  TCAGCCACAGTCTGCTTCAG |
| myxovirus (influenza virus) resistance 1, interferon-inducible protein (MX1) | NM_173940 | F  R | CAGGTGGAAAAGGAAATCAG  CAGGAAGGTCTATCAGGGTCA |
| 2',5'-oligoadenylate synthetase 1, 40/46kDa (OAS1Y) | NM_001040606 | F  R | AGTTCTCCCCCTGCTTCAC  GCTGCTCCTTACACAGTTGG |
| radical S-adenosyl methionine domain containing 2 (RSAD2) | NM_001045941 | F  R | GGCTTCTGCTTCCACACA  TTCTCCATACCTGCTTCTTTCA |
| Cyclic GMP-AMP Synthase (CGAS) | XM_005210662 | F  R | TACGAGCGAGTGAAGGTTCC  TACGAGCGAGTGAAGGTTCC |
| ADAM Metallopeptidase With Thrombospondin Type 1 Motif 20 (ADAMTS20) | NM_001206093 | F  R | GACTCATCCCAGTGTGCAGG  CAGCACAGGGCATACTCGAT |
| HECT And RLD Domain Containing E3 Ubiquitin Protein Ligase 5 (HERC5) | NM_001101995 | F  R | GCTCACAAAGAGTGGACTGGT  CCAGTTACCAAACGGGGTCT |
| Interferon Alpha Inducible Protein 6 (IFI6) | NM_001075588 | F  R | TCCTCCAAGATACGGTGACAAAG  GCCGCAGGTGTAGAGTAGC |
| Interferon Induced Protein 44 (IFI44) | XM_002686295 | F  R | GCTCACGCATGTGGATACCT  TGTGAACTGCCTCTAGCTGAA |
| Nectin Cell Adhesion Molecule 3 (NECTIN3) | ENSBTAT00000032332* | F  R | ACCAGATTTGCCAAAGGAAGGA  CCATCTAACCTGCTCCACACG |
| Prune Homolog 2 With BCH Domain (PRUNE2) | ENSBTAT00000017205* | F  R | ACTGAGGTAACTGCAGTCCG  AACTGACGTGGTCTCACACG |
| Sterile Alpha Motif Domain Containing 9 (SAMD9) | ENSBTAT00000061545* | F  R | AACTCGGAGAAAGGTGGCTG  CCATGGTGTGTACAGCGTCT |
| Slit Guidance Ligand 2 (SLIT2) | NM_001191516 | F  R | GGTTGGAGCAGAGGAACGAG  TCAAATCCAATCTCTCGGTGTTG |
| TNF Superfamily Member 10 (TNFSF10) | XM_002684917 | F  R | AGGGTCCTAAGAGGGTAGCTG  ATTCTTGGAGCCTGGAACTGG |
| Wnt Ligand Secretion Mediator (WLS) | NM_001192651 | F  R | GAAGACCAATCCAACGGTGA  ACAGAGCGTTCGTTGGCAT |
|  |  |  |  |
|  |  |  |  |

# Table S1. Details of oligonucleotide primers used in qRT-PCR. F and R denote forward and reverse primers respectively. *Primers designed based on Ensembl transcript.
