# Supplementary material for: Susceptibility to disease (tropical theileriosis) is associated with differential expression of host genes that possess motifs recognised by a pathogen DNA binding protein
Source: PLoS One. 2022 Jan 21;17(1):e0262051. doi: 10.1371/journal.pone.0262051 (PMC8782480; doi:10.1371/journal.pone.0262051)
Supplement: S2 Table — Rows denote RNA-seq sample set derived from 6 Sahiwal (S1-6) or 5 Holstein (H1-5) infected cell lines. Columns denote the % of reads mapped to the B. taurus genome. Summary denotes mean % of reads mapped, standard deviation and no significant difference between means. (DOCX) [file pone.0262051.s005.docx]

**Table S3. Overall read alignment rate of RNA-seq reads of Sahiwal (S1-S6) and Holstein (H1-H5) infected cell lines to *B. taurus* reference genome.**

S1: 50.91% H1: 49.37%

S2: 49.47% H2: 49.97%

S3: 49.91% H3: 49.42%

S4: 48.23% H4: 50.49%

S5: 49.58% H5: 49.10%

S6: 49.18%

**Summary**

49.55 +/-0.88 vs 49.67 +/-0.56 (i.e. S1-6 vs H1-5), T test (P = 0.40) – no significant difference
