# Supplementary material for: Susceptibility to disease (tropical theileriosis) is associated with differential expression of host genes that possess motifs recognised by a pathogen DNA binding protein
Source: PLoS One. 2022 Jan 21;17(1):e0262051. doi: 10.1371/journal.pone.0262051 (PMC8782480; doi:10.1371/journal.pone.0262051)
Supplement: S3 Table — In total 655 upstream regulators were found with a p-value of < 0.05; including 123 with a significantly predicted activation state (activated or inhibited, based on a z-score >2, or <-2). Only the top 20 activated and top 20 inhibited regulators based on z-score are presented in the table. (DOCX) [file pone.0262051.s006.docx]

**Table S7: Top upstream regulators with predicted activated or inhibited activation states in Infection Associated Genes.** In total 655 upstream regulators were found with a p-value of < 0.05; including 123 with a significantly predicted activation state (activated or inhibited, based on a z-score >2, or <-2). Only the top 20 activated and top 20 inhibited regulators based on z-score are presented in the table.

| **Upstream Regulator** | **Molecule Type** | **Predicted Activation State** | **Activation z-score** | **p-value of overlap** | **Genes in dataset (Number of regulators from data in network)** |
| --- | --- | --- | --- | --- | --- |
| poly rI:rC-RNA | biologic drug | Inhibited | -4.407 | 2.93E-07 | 105 (15) |
| IFNA2 | cytokine | Inhibited | -4.386 | 1.85E-11 | 84 (15) |
| IRF7 | transcription regulator | Inhibited | -4.172 | 6.14E-16 | 86 (14) |
| IRF3 | transcription regulator | Inhibited | -4.146 | 2.96E-09 | 86 (14) |
| IFN Beta | group | Inhibited | -4.03 | 1.71E-08 | 78 (14) |
| Interferon alpha | group | Inhibited | -3.948 | 8.52E-20 | 101 (15) |
| IFNL1 | cytokine | Inhibited | -3.915 | 3.74E-12 | 155 (15) |
| CpG ODN 2006 | chemical reagent | Inhibited | -3.85 | 1.21E-09 | 132 (16) |
| IFNB1 | cytokine | Inhibited | -3.824 | 1.36E-09 | 90 (13) |
| IFNG | cytokine | Inhibited | -3.678 | 3.44E-10 | 152 (14) |
| lenalidomide | chemical drug | Inhibited | -3.543 | 2.47E-03 | 38 (4) |
| IRF5 | transcription regulator | Inhibited | -3.523 | 6.33E-10 | 85 (17) |
| Ifnar | group | Inhibited | -3.492 | 1.73E-10 | 118 (18) |
| IFNA1/IFNA13 | cytokine | Inhibited | -3.377 | 2.14E-09 | 136 (16) |
| TLR9 | transmembrane receptor | Inhibited | -3.317 | 3.74E-07 | 142 (15) |
| EIF2AK2 | kinase | Inhibited | -3.266 | 1.37E-06 | 89 (13) |
| IRF1 | transcription regulator | Inhibited | -3.207 | 4.29E-09 | 157 (19) |
| imiquimod | chemical drug | Inhibited | -3.154 | 4.76E-05 | 97 (17) |
| MAVS | other | Inhibited | -3.12 | 2.20E-06 | 85 (12) |
| PRL | cytokine | Inhibited | -3.105 | 6.48E-10 | 138 (15) |
| MAPK1 | kinase | Activated | 4.318 | 4.46E-09 | 136 (14) |
| TRIM24 | transcription regulator | Activated | 4.308 | 2.54E-12 | 49 (7) |
| PNPT1 | enzyme | Activated | 3.742 | 1.86E-12 | 131 (11) |
| SIRT1 | transcription regulator | Activated | 3.445 | 6.27E-04 | 177 (16) |
| NKX2-3 | transcription regulator | Activated | 3.359 | 8.59E-05 |  |
| SOCS1 | other | Activated | 3.117 | 2.38E-04 | 130 (15) |
| VEGFA | growth factor | Activated | 3.057 | 2.63E-03 | 135 (10) |
| SP110 | transcription regulator | Activated | 3.051 | 1.23E-05 |  |
| ACKR2 | G-protein coupled receptor | Activated | 3 | 2.81E-07 |  |
| POR | enzyme | Activated | 2.897 | 4.44E-07 |  |
| NUPR1 | transcription regulator | Activated | 2.84 | 1.35E-01 |  |
| IKZF3 | transcription regulator | Activated | 2.828 | 1.45E-04 |  |
| INSIG1 | other | Activated | 2.727 | 2.34E-06 | 90 (7) |
| IL1RN | cytokine | Activated | 2.714 | 3.74E-10 | 145 (15) |
| geldanamycin | chemical - endogenous non-mammalian | Activated | 2.646 | 1.71E-02 |  |
| USP18 | peptidase | Activated | 2.592 | 1.69E-06 | 79 (14) |
| TAB1 | enzyme | Activated | 2.449 | 5.56E-05 | 83 (11) |
| DNASE2 | enzyme | Activated | 2.407 | 4.99E-07 |  |
| sterol | chemical - endogenous mammalian | Activated | 2.407 | 2.49E-05 | 72 (7) |
| IL10RA | transmembrane receptor | Activated | 2.4 | 4.94E-06 | 125 (11) |
