# Supplementary material for: Susceptibility to disease (tropical theileriosis) is associated with differential expression of host genes that possess motifs recognised by a pathogen DNA binding protein
Source: PLoS One. 2022 Jan 21;17(1):e0262051. doi: 10.1371/journal.pone.0262051 (PMC8782480; doi:10.1371/journal.pone.0262051)
Supplement: S10 File — Green highlight designates genes that display differential expression between Sahiwal and Holstein infected cells. (DOCX) [file pone.0262051.s016.docx]

**S16. List of genes in integrin signalling pathway that bear different numbers of nucleotide motif bound by TashAT2 in *B. taurus* and *B. indicus* genomes.** Green highlight designates genes that display differential expression between Sahiwal and Holstein infected cells.

MAPK10

LAMA2

LAMC1

BRAF

ITGA8

PIK3C3

DOCK1

ITGB8

COL9A1

VCL

ITGA4

COL11A1

RAPGEF1

ACTN2

COL4A4

ELMO1

SOS2

COL14A1

FLNB

RHOA

LAMA1

ITGB5

COL3A1

ITGAV

ITGA6

RAP1A

ARHGAP26

COL27A1

COL12A1

FYN

COL4A1

LAMA5

PIK3C2A

PIK3C2B

PIK3CD

ARHGAP10

PIK3R1

ITGA1
