# Supplementary material for: Susceptibility to disease (tropical theileriosis) is associated with differential expression of host genes that possess motifs recognised by a pathogen DNA binding protein
Source: PLoS One. 2022 Jan 21;17(1):e0262051. doi: 10.1371/journal.pone.0262051 (PMC8782480; doi:10.1371/journal.pone.0262051)
Supplement: Attachment — Submitted filename: Shiels-etal.resubmission.docx [file pone.0262051.s018.docx]

Susceptibility to disease (tropical theileriosis) is associated with differential expression of host genes that possess motifs recognized by a pathogen DNA binding protein

Author: Shiels et al.

This is a resubmission of a paper in which authors compare patterns of gene expression in ex vivo Theileria annulata-infected bovine cells lines from cattle breeds susceptible or tolerant to T. annulata infection to identify host and parasite factors that impact the susceptibility phenotype. The authors found plausible links between differences in tolerance and interferon-stimulated gene pathways, production of INFB1, inflammatory responses and oncogenesis. Critically, they identify a motif bound by a parasite gene that is differently distributed in the genome of both breeds. The authors find that genes with differences between breeds in the presence/absence/number of these motifs were enriched for functions potentially connected to infection.

This is a well-written paper with several interesting findings, leading to new hypotheses related to interactions between host and parasite, as well as infection susceptibility.

This resubmission addresses my previous concerns adequately.

Some minor issues:

- Pls add a first line to all supplemental word and xls tables with the title of the table
- Lack of consistency throughout text in use of versus, vs, vs. (best to use an italicized form, as *vs*.)
- L136, word missing in “offer the best available for“
- L191: change “mapped to the bovine” to “mapped to the Bos taurus”
- L319: change “number of reads” to “proportion of reads” (also, see typo in table S3)
- L629-630” “Subsequent PANTHER analysis showed”. Which genes were fed to this analysis? All 11,983 from B. taurus? Only the 7,575 shared? Those not shared? ...? The interpretation of the enrichment analysis will be affected.
- Fig 7C. I suggest using in Panel C different colors from those in legend in panel B
- L707-708: “This result may indicate that a number of infection-associated genes differentially expressed between breeds have been missed by our analysis “ I am not sure I follow. Couldn’t there be constitutively expressed genes, differentially expressed between breeds, regardless of infection status?
