# Supplementary material for: Susceptibility to disease (tropical theileriosis) is associated with differential expression of host genes that possess motifs recognised by a pathogen DNA binding protein
Source: PLoS One. 2022 Jan 21;17(1):e0262051. doi: 10.1371/journal.pone.0262051 (PMC8782480; doi:10.1371/journal.pone.0262051)
Supplement: Attachment — Submitted filename: Shiels Response to reviewers_2.docx [file pone.0262051.s019.docx]

PONE-D-21-24458R1

Susceptibility to disease (tropical theileriosis) is associated with differential expression of host genes that possess motifs recognised by a pathogen DNA binding protein

PLOS ONE

**Response to reviewers,**

Thanks to the reviewers for making the effort to look at our manuscript for a second time. We were pleased that the three reviewers indicated that we had adequately answered all previous concerns.

In response to minor issues raised by Reviewer 3, we have made the following amendments/corrections

**Reviewer 3.**

This resubmission addresses my previous concerns adequately.

Some minor issues:

- Pls add a first line to all supplemental word and xls tables with the title of the table
- Response – edited as requested
- Lack of consistency throughout text in use of versus, vs, vs. (best to use an italicized form, as *vs*.)
- Response - corrected to *vs.* throughout
- L136, word missing in “offer the best available for“
- Response – edited to “offer the best infected cell lines available….”
- L191: change “mapped to the bovine” to “mapped to the Bos taurus”
- Response – corrected to “mapped to the *B. taurus*
- L319: change “number of reads” to “proportion of reads” (also, see typo in table S3)
- Response – change as requested
- L629-630” “Subsequent PANTHER analysis showed”. Which genes were fed to this analysis? All 11,983 from B. taurus? Only the 7,575 shared? Those not shared? ...? The interpretation of the enrichment analysis will be affected.
- Response – edited to “Subsequent PANTHER analysis of the shared genes data set showed an enrichment”
- Fig 7C. I suggest using in Panel C different colors from those in legend in panel B
- Response - colours in panel C changed to Yellow and Grey
- L707-708: “This result may indicate that a number of infection-associated genes differentially expressed between breeds have been missed by our analysis “ I am not sure I follow. Couldn’t there be constitutively expressed genes, differentially expressed between breeds, regardless of infection status?
- We agree the logic of the text preceding this statement (i.e. that the pathways identified for both data sets are more or less the same) could be confusing, and the reviewer is correct that non-infection associated genes could be/are differentially expressed between breeds. Nevertheless, published data indicates we have missed candidates, so the text has been edited with “This result may indicate” deleted. The text now reads – “However, previous studies indicate that a number of infection-associated genes differentially expressed between breeds have been missed by our analysis, and there are at least several candidates.”
